# Optimized Extraction of Polyphenols from Kiwifruit Peels and Their Biological Activities

**DOI:** 10.3390/biotech13040054

**Published:** 2024-12-02

**Authors:** Batoul Shkeir, Nada El Darra, Bilal Azakir, Salma Khazaal, Elie Salem Sokhn, Mohamed Koubaa, Richard G. Maroun, Nicolas Louka, Espérance Debs

**Affiliations:** 1 Department of Nutrition and Dietetics, Faculty of Health Sciences, Beirut Arab University, Tarik El Jedidah, Riad El Solh, Beirut 1107 2809, Lebanon; batoulshkeir673@gmail.com (B.S.); n.aldarra@bau.edu.lb (N.E.D.); s.khazaal@bau.edu.lb (S.K.); 2 Molecular and Translational Medicine Laboratory, Faculty of Medicine, Beirut Arab University, Beirut 1107 2809, Lebanon; b.azakir@bau.edu.lb; 3 Department of Medical Laboratory Technology, Faculty of Health Sciences, Beirut Arab University, Beirut 1107 2809, Lebanon; e.sokhn@bau.edu.lb; 4 Université de Technologie de Compiègne, ESCOM, TIMR (Integrated Transformations of Renewable Matter), Centre de Recherche Royallieu-CS 60319, 60203 Compiègne CEDEX, France; 5 Centre d’Analyses et de Recherche, Unité de Recherche Technologies et Valorisation Agro-alimentaire, Faculté des Sciences, Université Saint-Joseph de Beyrouth, Mar Roukos, Dekwaneh, P.O. Box 1514, Riad El Solh, Beirut 1107 2050, Lebanon; richard.maroun@usj.edu.lb (R.G.M.); nicolas.louka@usj.edu.lb (N.L.); 6 Department of Biology, Faculty of Arts and Sciences, University of Balamand, P.O. Box 100, Tripoli 1300, Lebanon

**Keywords:** kiwi peels, infrared-assisted extraction, optimization, polyphenols, antioxidant, antibacterial, anticancer

## Abstract

(1) Background: Kiwifruit is a globally valued fruit. Its industrial processing produces a substantial amount of waste, particularly peels, which present an appealing potential source of bioactive compounds. This study focuses on optimizing the extraction of phenolics from kiwi peels using a water bath (WB) and infrared irradiation (IR) and assessing their biological activities. (2) Methods: Optimal conditions for polyphenol extraction from kiwifruit peels, in terms of temperature and time, were determined using Response Surface Methodology. Total phenolic content (TPC) was measured by the Folin–Ciocalteu method, and antioxidant activity was assessed utilizing the DPPH method. Antibacterial activities against *Bacillus cereus*, *Staphylococcus aureus*, *Escherichia coli*, and *Salmonella* Typhimurium were determined using the minimum inhibitory concentration (MIC). The lyophilized extract was tested for its anticancer effect on A549 lung cancer cell lines. The phytochemical profiles of the WB and IR extracts were analyzed through High-Performance Liquid Chromatography (HPLC). (3) Results: The optimal extraction conditions for the WB method were found to be 75 °C for 35 min, and 90 °C for 5 min for IR. The corresponding TPC obtained by IR was 21 mg GAE/g DM, which was 4.4 times higher than that obtained by WB (4.8 mg GAE/g DM). This indicates that IR was more efficient in extracting phenolics from kiwi peels. The antioxidant activity under the optimum conditions of WB and IR was 14 mg TE/g DM and 16 mg TE/g DM, respectively. Both the WB and IR extracts demonstrated antibacterial activity against *B. cereus* with an MIC value of 25 mg/mL. Additionally, the IR extract displayed an antibacterial effect against *S. aureus*, with an MIC value of 50 mg/mL. The WB and IR kiwi peel extracts were effective in significantly reducing A549 cell viability at 50 µg/mL and 100 µg/mL, respectively. Caffeic acid (0.54 ppm) and catechin (5.44 ppm) were the major polyphenols identified in WB and IR extracts, as determined by HPLC. (4) Conclusions: IR was more efficient in extracting phenolics from kiwi peels than WB. The findings also suggest that kiwi peel can be effectively utilized as an antioxidant, antibacterial, and anticancer agent.

## 1. Introduction

The kiwifruit, generally referred to as “the monarch of fruits”, has emerged as one of the most significant fruits in the world in recent years [1]. Native to north-central China, kiwifruit is a product of the *Actinidia* plant genus, which includes roughly 60 species [2]. China and Italy are the top producers of *Actinidia deliciosa “Hayward”*, which is the most widely grown plant in this genus [3]. Kiwifruit offers numerous health benefits, including asthma relief, immune system strengthening, diabetes control, cancer prevention, skin repair, anemia treatment, and heart health improvement [4]. However, allergic reactions, such as rashes, stomach pains, and anaphylactic shock, may occur due to actinidin protein, a common allergen [5]. Due to their large fruit size, green flesh color, delightful flavor, high vitamin C content, and long shelf life, “Hayward” kiwis are the top choice for many Chinese customers [6].

Kiwifruit is rich in carbohydrates (fibers and sugars), proteins, vitamins, minerals, and polyphenols [7]. It is generally consumed fresh, but it can also be utilized in the production of wine, spirits, syrups, lyophilized goods, juices, jam, yogurt, ice cream, and other popular items [1,2]. Therefore, the kiwifruit generates a common by-product, the peels, which are rich in phenolic compounds such as flavonoids and polyphenols, with potential applications in food, pharmaceutical, and medical industries [2,8,9,10]. Plant extracts and phenolic compounds are recognized for their anti-inflammatory, antimicrobial, anticancer, and anti-aging activities, as well as their ability to defend against inflammation and oxidative stress caused by airborne particulate matter [11]. The three major polyphenols present in kiwifruit are caffeic, protocatechuic, and vanillic acids [12]. Hydroxybenzoic acid (2.75–382.78 mg/kg), gallic acid (12.83–334.03 mg/kg), and syringic acid (10.05–224.31 mg/kg) were found in the peels of kiwifruits [13]. According to Alim et al. (2019), the primary phenolic compounds detected in kiwifruit peels were catechin, epicatechin, epigallocatechin, and quercetin, with approximately 29%, 16%, 5%, and 45%, respectively [14].

Numerous factors, particularly fruit species, gardening practices, soil type, growing area, storage ripening conditions, and fruit maturity, may have an impact on the phenolic composition of kiwifruit [15]. The phenolic content and the antiradical activity of many plants’ extracts were evaluated using various solvents and extraction techniques [16,17,18]. Various extraction techniques have been employed to extract bioactive compounds from plant extracts and by-products [19,20,21,22]. The common methods for extracting phenolic compounds include Soxhlet extraction [23], ultrasound-assisted extraction [24,25], microwave-assisted extraction [25], and infrared-assisted extraction [26,27,28], with the usage of various solvents including acetone, ethanol, methanol, and acetonitrile, or by mixing them with water [29,30]. Additionally, the yield of phenolic content is linked to factors such as the temperature and extraction time [16,31,32].

This study aims to set the optimal conditions for the extraction of kiwifruit peels and to assess the biological activities of the extracted polyphenols. The extraction methods employed include water bath (WB) and infrared-assisted irradiation (IR). To accomplish this, optimization was performed utilizing response surface methodology (RSM), which has several benefits, including requiring fewer experimental measurements, interpreting the data statistically, and identifying any interactions between variables [15,33,34,35]. This study, to our knowledge, is the first to evaluate the polyphenol content and biological activities of WB and IR kiwi peel extracts (KPEs).

## 2. Materials and Methods

### 2.1. Raw Material

Kiwi (*Actinidia deliciosa*) Hayward, produced in Turkey, was purchased from the central fruit and vegetable market in Beirut. All kiwifruits were peeled and then dried for 24 h at 50 °C in a hot air oven. The dried peels were stored at room temperature in an amber nylon bag.

### 2.2. Chemicals, Reagents, and Media

All the chemicals and reagents required for the experiments, comprising gallic acid, Folin–Ciocalteu reagent, sodium carbonate (Na_2_CO_3_), Trolox (6-hydroxy-2,5,7,8-tetramethylchroman-2-carboxylic acid), DPPH (2,2-diphenyl-1-picryllhydrazyl), and HPLC standards, were obtained from Sigma-Aldrich (Steinem, Germany). Dulbecco’s Modified Eagle Medium (DMEM), dimethyl sulfoxide (DMSO), phosphate buffer saline, MTT reagent (3-(4,5-dimethylthiazol-2-yl)-2,5-diphenyltetrazolium bromide), fetal bovine serum (FBS), Roswell Park Memorial Institute (RPMI) were purchased from Sigma-Aldrich (Darmstadt, Germany). Penicillin-streptomycin was purchased from Biowest (Nuaillé, France). Mueller–Hinton Broth (MHB), Mueller–Hinton Agar (MHA), and MacConkey Agar (MAC) were purchased from HIMEDIA (Mumbai, India).

### 2.3. Dry Matter of Kiwi Peels

The dry matter (DM) content of kiwi peels was determined by placing the peels in a hot air oven for 24 h at 105 °C. The dry matter content was then calculated, and the result was expressed as a percentage of the total peel weight. The dry matter content of kiwi peels was found to be 20.57% *w*/*w*.

### 2.4. Extraction Methods

#### 2.4.1. Extraction Procedure

A range of solid-to-liquid ratios, from 1/10 to 1/30 g/mL, was tested for the extraction process at a certain temperature and time. Once the solid-to-liquid ratio yielding the highest total phenolic content (TPC) was determined, the RSM was applied to optimize the extraction conditions. The process involved using 60 mL of water as a solvent and adding 3 g of kiwi peel powder at a solid-to-liquid ratio of 1/20 g/mL. Following filtration, the resulting filtrates were centrifuged at 5000 rpm for 10 min. The filtrates were then stored at −18 °C until used.

#### 2.4.2. Water Bath Extraction

In the water bath extraction (WB) process, kiwi peels were added to a flask containing preheated distilled water and placed in a water bath (DKZ-1 series, (Shanghai Lilang Scientific Instrument Co., Ltd., Shanghai, China) at a certain temperature for a specified duration.

#### 2.4.3. Infrared-Assisted Extraction

The infrared extraction (IR) setup was patented (Patent No: 2017-12 11265L) and developed through a collaboration between the Faculty of Sciences at Saint Joseph University of Beirut and the Faculty of Arts and Sciences at the University of Balamand. The apparatus features a flat ceramic infrared emitter connected to a control system that utilizes proportional–integral–derivative (PID) to regulate the temperature (Figure S1 [36]). The kiwi peels were subsequently placed in distilled water in a round-bottom flask, sealed with aluminum foil, and left for a specific duration at a certain temperature. The flask was positioned approximately 1 cm away from the IR emitter.

### 2.5. Experimental Design

Various factors influence the quantity and quality of total polyphenols. The extraction processes were optimized using RSM to assess the influence of every parameter as well as their combined effects. In the current study, RSM considered the temperature “T” and the extraction time “t”. To assess the effect of the temperature and time of extraction on the total phenolic content and concentration of DPPH, a central composite design experiment was set up. The experiment consisted of twelve runs with four repetitions of the central point. The temperature varied from 45 to 75 °C, and the extraction time from 20 to 60 min. The highest and lowest levels of these variables were coded as +1 and −1, respectively. This design was employed for both extractions using WB and IR.

Considering two experimental parameters and one response, the data were fitted to attain a second-degree regression equation as follows:

Y = α_0_ + α_1_ × T + α_2_ × t + α_3_ × T^2^ + α_4_ × T × t + α_5_ × t^2^

In this context, “Y” represents the predicted response factor; α_0_ denotes the average response value at the central point; α_1_ and α_2_ refer to the linear coefficients; α_3_ and α_5_ correspond to the quadratic coefficients; and α_4_ denotes the coefficient for interaction. 

### 2.6. Determination of Total Phenolic Content

TPC was measured using the Folin–Ciocalteu method, as explained in a previous study [37]. In this process, to 500 μL of the Folin–Ciocalteu reagent (diluted 1/10 *v*/*v*) and 400 μL of Na_2_CO_3_ 7.5% (*w*/*v*), 100 μL of the kiwi peel liquid extract was added. The resulting mixture was incubated for 10 min at 60 °C, and then at 4 °C for 10 min. The absorbance was recorded at 750 nm utilizing a UV–Vis spectrophotometer (GENESYS 10 UV, Thermo Electron Corporation, Waltham, MA, USA). Quantification was carried out using a gallic acid calibration curve, with the results expressed as milligrams of gallic acid equivalent per gram of dry matter (mg GAE/g DM).

### 2.7. Determination of Antioxidant Activity

The antioxidant activity was determined by evaluating the capability of phenolic compounds in the extracts to reduce the DPPH (2,2-diphenyl-1-picrylhydrazyl) free radical [38]. To summarize, 1.45 mL of DPPH (0.06 mM) was mixed with 50 μL of KPE or the positive control, Trolox. After being kept at room temperature for 30 min in the dark, the absorbance was recorded at 515 nm, where the blank was pure methanol. The DPPH free radical inhibition percentage was determined using the following formula:

$$Inhibition\;percentage=\frac{Absorbance\;of\;negative\;control-Absorbance\;of\;sample\;}{Absorbance\;of\;negative\;control}\times 100$$

The antioxidant activity of KPEs was measured in micrograms of Trolox equivalent per gram of dry matter (mg TE/g DM).

### 2.8. Antibacterial Activity Assay

#### 2.8.1. Bacterial Strains

The antibacterial activity of KPEs was assessed on Gram-positive (*Bacillus cereus* and *Staphylococcus aureus* ATCC25923) and Gram-negative bacteria (*Escherichia coli* ATCC25922 and *Salmonella* Typhimurium ATCC14028). The strains were supplied by the Faculty of Health Sciences, Medical Lab Department at Beirut Arab University. They were kept in glycerol broth and stored at −18 °C until needed for subsequent use.

#### 2.8.2. Inoculum Standardization

The preserved bacterial strains were cultured on specific agar types: MAC for Gram-negative bacteria and MHA for Gram-positive bacteria, to obtain fresh colonies. These isolated bacterial colonies were then transferred from a fresh culture to sterile test tubes containing 0.9% sterile saline to achieve 0.5 McFarland (10^8^ CFU/mL).

#### 2.8.3. Determination of the Minimum Inhibitory Concentration

The minimum inhibitory concentration (MIC) of KPEs was determined utilizing the broth microdilution technique [39]. The bacterial suspension was adjusted to a 0.5 McFarland standard. Next, 1 mL of MHB was transferred to 6 tubes. Subsequently, 1 mL of the KPE, initially at a concentration of 50 mg/mL, was transferred to the original tube, followed by serial dilution to concentrations of 25, 12.5, 6.25, 3.1, and 1.55 mg/mL in the subsequent tubes. Then, 1 mL of the bacterial suspension (10^8^ CFU/mL) was aseptically transferred to every tube. The bacterial suspension and the broth were considered as the positive control, while the broth and broth with extracts served as the negative control. After incubation for 24 h at 37 °C, the MIC was identified as the lowest concentration that prevented bacterial growth.

### 2.9. Determination of the Anti-Tumor Activity In Vitro 

#### 2.9.1. Cell Culture and Treatment

The anti-tumor activity of the lyophilized KPEs was evaluated against the A549 lung cancer cell line, generously provided by Professor Salem Chouaib (Thumbay Research Institute for Precision Medicine, Gulf Medical University, Ajman, UAE). The cells were cultured in RPMI-1640, enriched with 10% heat-inactivated FBS and 1% penicillin/streptomycin, and were then incubated at 37 °C in a 5% CO_2_ atmosphere.

#### 2.9.2. MTT Assay

The anti-tumor effectiveness of the KPEs was assessed in vitro using 3-(4,5-dimethylthiazol-2-yl)-2,5-diphenyltetrazolium bromide (MTT) assay as per the instructions provided by the manufacturer [14,40]. In this study, 100 μL of 7 × 10^3^ cells per well were seeded in a 96-well plate and then incubated at 37 °C for 24 h. After 24 h of incubation, different working solutions with various concentrations (10, 25, 50, 75, 100, 150 μg/mL) were prepared from a fresh stock solution (1 mg/mL) of lyophilized KPEs. Subsequently, 100 μL of the different working solutions was added to each well as a treatment for the seeded cells. Control wells consisted of cells with their specific media (DMEM), while the mock ones contained cells and media with 0.06% DMSO. After 72 h of treatment with the extracts, the medium was pipetted out from the wells and then replaced with 100 μL of 10% MTT solution for 4 h. The MTT was removed, 50 μL of DMSO was transferred to each well to solubilize the formazan crystals, and the absorbance was recorded with an ELISA reader (Thermo Fisher Scientific, Boston, MA, USA) at 620 nm. The results were expressed as cell viability (%) according to the following equation:

$$Cell\;viability\;\%=\frac{At-Ab}{Am}\times 100$$

where *At* is the average absorbance of tested samples, *Ab* is the average absorbance of the blank, and *Am* is the average absorbance of the mock.

### 2.10. High-Performance Liquid Chromatography Analysis

The phenolic compounds in KPEs were identified and quantified by high-performance liquid chromatography (HPLC) at the Lebanese Agricultural Research Institute (LARI), Fanar, Lebanon, following the method detailed by Vizzotto et al. (2007) [40]. An Agilent 1100 series HPLC system (Teknokroma Professional Friendly Lichrospher 100 RP18 5 mM, 25 × 0.46, Serial number NF-21378, Barcelona, Spain), a Zorbax column oven (Barcelona, Spain), an autosampler, and a diode array detector were used for this analysis. A C18 column (250 × 4.6 mm; 5 μm) was employed for the separation of phenolic compounds. The standards utilized included: caffeic acid, catechin, chlorogenic acid, ellagic acid, gallic acid, hydroxybenzoic acid, *p*-coumaric acid, protocatechuic acid, quercetin, trans-cinnamic acid, and rutin. The injection volume was fixed at 10 μL, and the flow rate was kept at 1 mL/min. The mobile phase comprised of acidified nanopure water at pH 2.3 with HCl (A) and methanol (B) of HPLC grade. Under isocratic conditions, elution began with 85% acidified nanopure water with HCl and 15% methanol from 0 to 5 min. A gradient profile was utilized over 5 to 30 min, shifting from 85% A and 15% B to 0% A and 100% B. Isocratic conditions were then maintained from 30 to 35 min with 0% A and 100% B. Phenolic compounds were detected by matching the peaks’ retention times with known standards. To quantify their concentrations, standard curves were generated for each compound by utilizing various concentrations of the corresponding standard solutions.

### 2.11. Statistical Analysis

Statistical analysis included unpaired t-tests, and the data were analyzed utilizing STATGRAPHICS^®^ Centurion XVI.I (Statgraphics 18, The Plains, VA, USA) software to optimize the extraction process.

## 3. Results and Discussion

### 3.1. Determination of Solid-to-Liquid Ratio

Solid-to-liquid ratios including 1/10, 1/20, and 1/30 were evaluated by extraction at a fixed temperature and time (Table 1). Generally, when the quantity of solvent is increased, up to a certain extent, it results in a higher concentration gradient, allowing for the extraction of a greater amount of phenolic compounds [41]. In our case, it can be concluded that the quantity of solvent at 1/20 (g/mL) was enough to maximally extract the phenolic compounds using distilled water. Consequently, further increasing the amount of solvent did not affect the TPC yields. Similarly, Kehili et al. (2022) compared the extraction of defatted date seeds at 1/10, 1/20, and 1/30 solid-to-liquid ratios and chose 1/20 as the best ratio [42]. Hence, the solid-to-liquid ratio of 1/20 g/mL was chosen for the extraction processes, with a TPC of 12.78 ± 0.54 mg GAE/g DM.

### 3.2. Effect of Temperature and Time on TPC Yield and Antioxidant Activity (DPPH)

The RSM was employed to optimize the extraction of polyphenols. This was carried out to determine the optimal conditions for the WB and IR extraction methods to achieve maximum TPC and antioxidant activity. In this study, the solid-to-liquid ratio was fixed at 1/20 (g/mL) and the model was developed by varying the temperature and time. Table 2 provides the values of TPC (mg GAE/g DM) and DPPH (mg TE/g DM) for WB and IR KPEs. Concerning the several temperatures and time runs, the maximum TPC for WB and IR was 5.08 and 10.20 mg GAE/g DM, respectively. As for the antioxidant activity of KPEs, it was shown to be 10.50 and 9.49 mg TE/g DM for WB and IR, respectively.

**Figure 1:**
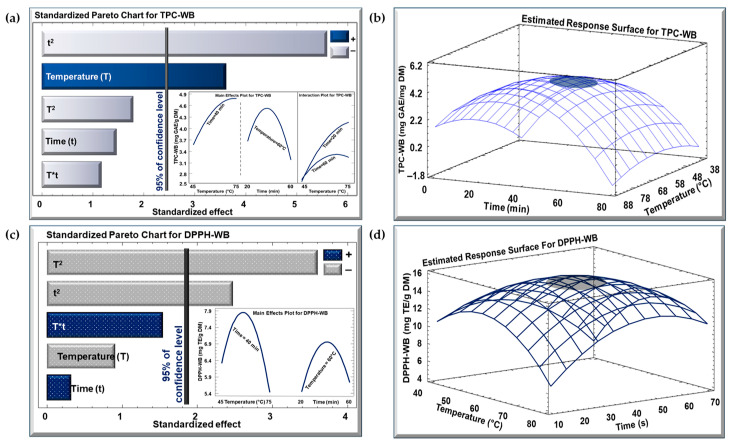
Standardized Pareto chart with insert for the effect of temperature and time for (**a**) TPC and (**c**) DPPH concentration for WB method, and estimated response surface for (**b**) TPC and (**d**) DPPH concentration. (+) denotes a positive effect, and (−) denotes a negative effect.

**Figure 2:**
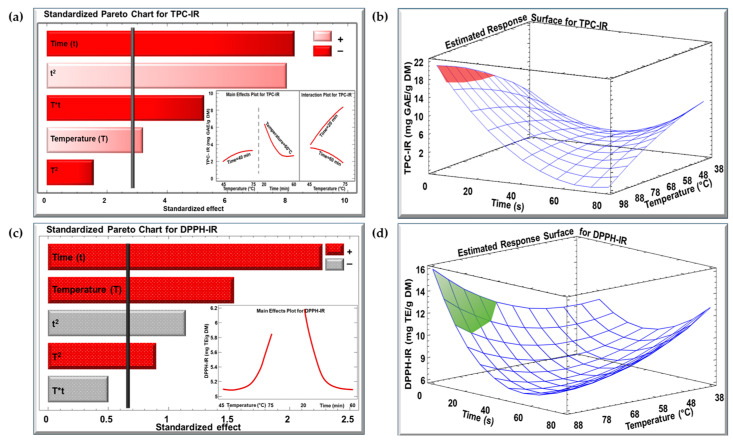
Standardized Pareto chart with insert for the effect of temperature and time for (**a**) TPC and (**c**) DPPH concentration for IR technique, and estimated response surface for (**b**) TPC and (**d**) DPPH concentration. (+) denotes a positive effect, and (−) denotes a negative effect.

In terms of the TPC and DPPH assay of KPEs, the impact of the studied factors (temperature and time) was examined utilizing the Pareto chart and the estimated response surface for the WB and IR techniques. The outcomes for the WB and IR extraction methods are demonstrated in Figure 1 and Figure 2, respectively. In the Pareto charts, a vertical bar implies a significant effect with a confidence level exceeding 95%.

The Pareto chart (Figure 1a) shows that temperature had a significant positive linear impact on the TPC of the WB extract (TPC-WB), as it increased from approximately 3.6 to 4.8 mg GAE/g DM between 45 and 75 °C (insert of Figure 1a). However, the significant quadratic effect of time negatively affected the TPC-WB, as it increased and then decreased to approximately 3.2 mg GAE/g DM, between 20 and 60 min (insert of Figure 1a). The optimal conditions for the TPC-WB are highlighted in Figure 1b, where any combination of temperature and time in the grey area yields the maximum TPC (almost 4.2 mg GAE/g DM). As for the Pareto chart (Figure 1c) of the DPPH of the WB extract (DPPH-WB), temperature and time had a significant negative quadratic impact on the DPPH-WB, where it decreased to 5.5 and 5.7 mg TE/g DM, respectively (insert of Figure 1c). The optimum conditions for the DPPH-WB are highlighted in Figure 1d, where any combination of temperature and time in the grey area yields the highest DPPH concentration (almost 14.8 mg TE/g DM).

The Pareto chart (Figure 2a) illustrates that temperature had a significant positive linear effect on the TPC of the IR extract (TPC-IR), increasing from about 2 to 3.5 mg GAE/g DM as the temperature rose from 45 to 75 °C (insert of Figure 2a). However, the quadratic effect of time had a significant negative impact, with TPC-IR decreasing from around 6.5 to 3 mg GAE/g DM between 20 and 60 min (insert of Figure 2a). Moreover, a significant interaction between temperature and time was observed for TPC-IR (insert of Figure 2a). At shorter extraction times (20 min), increasing the temperature from 45 to 75 °C increased TPC-IR. However, at longer times (60 min), this trend reversed, with higher temperatures leading to a decrease in TPC-IR. The optimal conditions for achieving the highest TPC-IR, nearly 20 mg GAE/g DM, are indicated in Figure 2b, where any combination of temperature and time in the red area produces the best results. In the case of the Pareto chart (Figure 2c) for the DPPH of the IR extract (DPPH-IR), temperature exhibited a strong positive linear and quadratic effect, increasing DPPH-IR from 5.1 to 5.9 mg TE/g DM (insert of Figure 2c). Time had both a positive linear and a negative quadratic effect, causing DPPH-IR to decrease from 6.2 to 5.1 mg TE/g DM. Figure 2d highlights the optimal conditions for DPPH-IR, where any temperature and time combination in the green area results in the highest DPPH concentration, reaching approximately 16 mg TE/g DM.

Based on various studies [43,44], raising the temperature enhances extraction yield by promoting mass transfer via enhanced solute solubility and diffusion coefficients. Elevating the extraction temperature, typically within the range of 20 to 80 °C, generally results in an increased yield of polyphenols [45]. A study by Rajha et al. (2014) demonstrated that raising the temperature from 40 to 80 °C produced a 2.7-fold increase in polyphenol extraction from grape pomace [46]. However, at higher temperatures, the potential for thermal degradation increases, leading to a reduction in polyphenol yield during high-temperature extractions [47]. It is worth noting that the temperature threshold for the degradation of specific polyphenols varies across different extraction studies [48]. This may account for the negative quadratic impact of temperature on DPPH concentration for WB, where, beyond a certain temperature (approximately 60 °C), the increase in polyphenol extraction was offset by their degradation. As for the negative quadratic effects of time, it could be attributed to the potential loss or damage of phenolic compounds when high temperatures are used for extended periods [48].

Table 3 displays the second-degree regression model formulas utilized for predicting the response values through statistical analysis.

### 3.3. Optimization of Extraction

**Figure 3:**
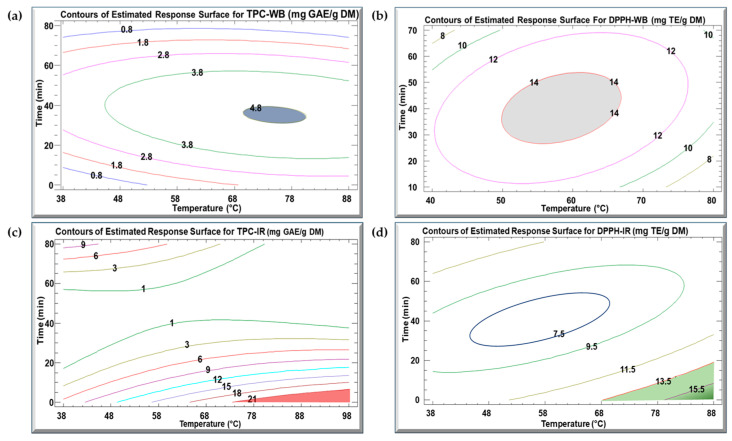
Contours of the estimated response surface for (**a**) TPC-WB, (**b**) DPPH-WB, (**c**) TPC-IR, and (**d**) DPPH-IR in the function of time and temperature for KPEs. The colored zone indicates the optimum conditions to attain the highest TPC and DPPH concentration.

Table 4 presents the optimal extraction conditions for both the WB and IR methods. The high R^2^ values, extending from 86.7 to 91.5%, demonstrate a reliable level of model performance. The contour plots in Figure 3 illustrate the estimated response surface for TPC and DPPH in relation to temperature and time for the extraction of kiwi peels by WB and IR. With different combinations of temperature and time, one TPC or DPPH value can be obtained. For the WB method, the optimal conditions for achieving a TPC value of 4.8 mg GAE/g DM (blue zone) were approximately 75 °C and 38 min (Figure 3a). Meanwhile, to attain the optimal DPPH concentration of 14.8 mg TE/g DM (grey zone) utilizing the WB method, the ideal conditions were around 58 °C and 40 min (Figure 3b). On the other hand, for the IR method, the optimal conditions for obtaining a TPC value of 21 mg GAE/g DM (light brown zone) were approximately 95 °C and 5 min (Figure 3c), while the optimal conditions for achieving a DPPH concentration of 16 mg TE/g DM (green zone) using the IR method were around 90 °C and 5 min (Figure 3d).

To validate the optimum values proposed by the model, kiwi peels were extracted by the WB and IR procedures using the predicted optimal parameters (Table 4). The WB extract acquired a TPC of 4.8 mg GAE/g DM, consistent with the predicted value. However, the observed values for TPC-WB, DPPH-WB, and DPPH-IR were to some extent lower than the predicted values, indicating that the model overestimated these results.

**Figure 4:**
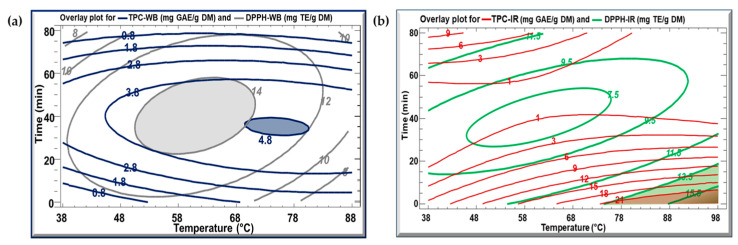
Overlay plots created from contours of estimated response surface for TPC and DPPH for (**a**) WB and (**b**) IR KPEs.

Figure 4 displays the overlay plot for the TPC and DPPH for the WB and IR extraction methods. In Figure 4a, the iso-response curves of TPC-WB (blue) and DPPH-WB (grey) are shown concurrently. The optimal extraction parameters for achieving the highest TPC (4.8 mg GAE/g DM) and DPPH concentration (14.8 mg TE/g DM) using the WB method can be selected from the overlapping region of the two zones, which corresponds to 70 °C for 35 min. In Figure 4b, the red and green zones represent the optimal extraction parameters for the IR method. As these zones overlap, the extraction parameters can be selected at 90 °C for 5 min to obtain a TPC value of 21 mg GAE/g DM and a DPPH concentration of 16 mg TE/g DM.

The TPC achieved through the IR method (21 mg GAE/g DM) was 4.4 times higher than that obtained through the WB method (4.8 mg GAE/g DM). The enhanced effectiveness of the IR method could be credited to the electromagnetic waves that activate the molecules by twisting, stretching, and bending modes [49]. Infrared irradiation is distinguished by its excellent heat transfer capability and its ability to directly penetrate the sample [50]. IR selectively raises the temperature of the sample matrix without affecting the surrounding air, potentially improving the extraction of bioactive molecules [49].

### 3.4. Antibacterial Activity

The antibacterial activity of WB and IR KPEs, recovered under optimum conditions (70 °C for 35 min for WB and 90 °C for 5 min for IR), was assessed by determining the minimum inhibitory concentration (MIC). The results indicated that the extracts exhibited inhibitory activity against Gram-positive bacteria, *B. cereus* and *S. aureus* (Figure S2), with no activity against Gram-negative ones (*E. coli* and *S. typhimurium*). Specifically, the results indicated that the WB KPE inhibited *B. cereus* at a MIC value of 25 mg/mL. Additionally, the IR KPE suppressed the growth of both *B. cereus* and *S. aureus* at MIC values of 25 and 50 mg/mL, respectively. These findings indicated that KPEs were more effective against Gram-positive bacterial strains than Gram-negative ones. This difference may be attributed to the higher activity of polyphenolic compounds on Gram-positive bacterial strains in comparison to Gram-negative bacteria. The lipopolysaccharide membrane present in Gram-negative bacteria acts as a barrier, limiting the uptake of these compounds. Moreover, bacterial mutations in porin proteins or efflux mechanisms may further reduce the activity of polyphenols against these organisms [51,52].

It is to be noted that the IR KPE showed inhibition against *S. aureus*, while the WB did not exhibit any effect. This result may be attributed to the efficiency of the IR technique in extracting a higher amount of polyphenols from kiwi peels, as previously mentioned. Moreover, this may be linked to the higher DPPH concentration in the IR KPE (16 mg TE/g DM) in comparison to the WB extract (14.8 mg TE/g DM), leading to more effective antibacterial activity.

### 3.5. Anti-Tumor Activity

**Figure 5:**
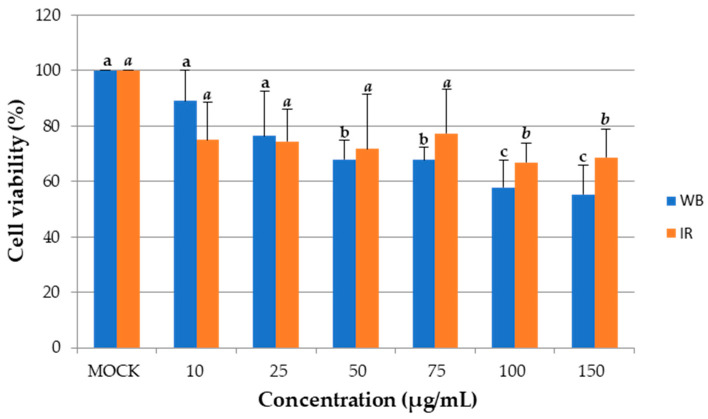
Anti-tumor activity of WB and IR KPEs on lung cancer cell line A549. Data represent the average of three different determinations ± SD. ^a,b,c^ Values sharing the same letter (normal for WB and italicized for IR) are not significantly different (*p* > 0.05).

The anti-tumor activity on A549 cells was observed for WB and IR KPEs, obtained under optimal conditions (70 °C for 35 min for WB and 90 °C for 5 min for IR), at different concentrations, as shown in Figure 5. The WB KPE effectively reduced A549 cell viability at 50 µg/mL by 28.34% and by 44.7% at 150 µg/mL. The IR KPE was effective in significantly reducing A549 cell viability to 42% at 100 µg/mL with no further significant reduction at 150 µg/mL.

According to prior studies [14,53], KPE was found to be effective in reducing cancer cells of HepG2-human liver and PANC-1-human pancreatic cancer cells by 75% viability at high dosages of 400 and 1000 μg/mL, respectively. Moreover, ElZawawy (2015) conducted a study demonstrating that the ethanol extract of kiwi peels exhibits 34.16% anti-tumor activity on the MCF-7 breast cancer cell line [54]. On the other hand, Zeinab (2018) [55] assessed that the anticancer activity of kiwi peels on MCF-2-breast and HepG2-human liver cancer cells showed no effect on cancer cell viability. The differences in the results could be ascribed to variations in kiwi cultivars and their phenolic composition, the type of solvent utilized for extraction, the sensitivity of the cancer cells, the concentrations of KPEs, and the length of the incubation period.

### 3.6. High-Performance Liquid Chromatography (HPLC) Analysis

HPLC analysis was performed to identify and quantify the phenolic compounds in the KPEs obtained under their optimal conditions from both the WB and IR methods. Caffeic acid (0.54 g/L) and catechin (5.44 g/L) were the primary polyphenols identified in the kiwi peel WB and IR extracts, respectively, through HPLC analysis (Figure S3). Caffeic acid, categorized as a hydroxycinnamic acid, demonstrates protective properties against chronic illnesses and cancer [56]. Catechins, which belong to the flavanol group, offer a wide range of health advantages, including anticancer, anti-obesity, anti-diabetic, anti-cardiovascular, anti-infectious, neuroprotective, and hepatoprotective impacts [57]. Hence, the previously detected biological activities could be attributed to those polyphenols. When kiwi peels were extracted by 80% acetone, caffeic acid and catechin were detected at 1.45 and 26.66 mg/100 g DM, respectively [55]. The variation may be due to differences in the kiwi fruit’s origin as well as the solvent and extraction methods utilized.

## 4. Conclusions

This study successfully demonstrated that kiwi peels, a by-product of industrial kiwifruit processing, could be transformed into a valuable source of bioactive compounds, particularly phenolics, through optimized extraction methods. The use of IR extraction proved to be significantly more efficient than WB, yielding a TPC that was 4.4 times higher under optimal conditions (21 mg GAE/g DM for IR compared to 4.8 mg GAE/g DM for WB). This difference highlighted the potential of IR extraction for maximizing the recovery of valuable compounds from kiwi peels. Moreover, IR extracts exhibited superior antioxidant activity (16 mg TE/g DM) compared to WB extracts (14 mg TE/g DM). Additionally, both methods produced extracts with antibacterial properties, particularly on Gram-positive bacteria such as *Bacillus cereus* and *Staphylococcus aureus*. The IR method further demonstrated enhanced antibacterial efficacy on *S. aureus* with a 50 mg/mL MIC. This suggested that IR extraction may offer broader antibacterial capabilities. Furthermore, the anticancer potential of KPEs was supported by the reduction in lung cancer (A549) cell viability, where the WB and IR KPEs were effective in significantly reducing cell viability at 50 µg/mL and 100 µg/mL, respectively. The phytochemical analysis revealed that catechin (5.44 ppm) was the major phenolic compound identified in the IR extract, while caffeic acid (0.54 ppm) was predominant in the WB extract, further emphasizing the distinct composition and potential health benefits of each extraction method. These findings suggested that kiwi peels, often regarded as waste, could be effectively repurposed for their valuable bioactive compounds, offering antioxidant, antibacterial, and anticancer properties. The study not only highlighted the advantages of IR extraction in terms of efficiency and biological activity but also highlighted the broader implications for sustainable waste management and the development of functional ingredients for use in food, pharmaceutical, and cosmetic industries. By utilizing kiwi peel waste, this research supported the move towards a circular economy, offering an eco-friendly and cost-effective strategy for the valorization of agricultural by-products.

## Figures and Tables

**Table 1:** Average total phenolic content (TPC) at various solid-to-liquid ratios.

Solid–Liquid Ratio (g/mL)	Average TPC (mg GAE/g DM)
1/10	10.99 ± 0.70
1/20	12.78 ± 0.54
1/30	12.80 ± 0.23

**Table 2:** Central composite design of independent variables and corresponding responses for TPC (mg GAE/g DM) and DPPH concentration (mg TE/g DM).

Runs	Variables		Responses			
	Temperature	Time	TPC		DPPH	
	(°C)	(min)	(mg GAE/g DM)		(mg TE/g DM)	
			WB	IR	WB	IR
1	45	20	2.82	2.44	8.32	6.32
2	75	20	4.10	8.25	10.44	9.49
3	45	60	2.94	3.94	10.50	6.68
4	75	60	3.13	3.52	8.59	6.76
5	39	40	2.49	2.11	7.51	5.62
6	81	40	4.86	10.20	9.86	4.77
7	60	11.8	2.56	4.58	8.01	5.72
8	60	68.2	1.77	2.82	6.97	5.91
9	60	40	4.63	3.32	7.74	5.31
10	60	40	3.95	3.27	5.30	5.87
11	60	40	4.38	2.05	4.08	4.81
12	60	40	5.08	3.09	5.43	5.93

**Table 3:** Second-order regression formulas for WB and IR methods.

TPC-WB=	−9.33 + 0.26 × T + 0.26 × t − 0.002 × T^2^ − 0.001 × T × t − 0.003 × t^2^
DPPH-WB=	32.70 − 0.88 × T + 0.078 × t − 0.009 × T^2^ + 0.003 × T × t − 0.003 × t^2^
TPC-IR=	7.10 + 0.45 × T − 0.15 × t − 0.002 × T^2^ − 0.005 × T × t + 0.005 × t^2^
DPPH-IR=	9.54 − 0.14 × T − 0.019 × t + 0.002 × T^2^ − 0.003 × T × t + 0.002 × t^2^

**Table 4:** Optimal extraction parameters for WB and IR techniques.

Parameters	Optimum Conditions			
	WB		IR	
	TPC	DPPH	TPC	DPPH
Temperature (°C)	75	58	95	90
Time (min)	38	40	5	5
TPC predicted value (mg GAE/g DM)	4.8	-	20.9	-
TPC observed value (mg GAE/g DM)	5	-	19.3	-
DPPH predicted value (mg TE/g DM)	-	14.8	-	16
DPPH observed value (mg TE/g DM)	-	13.7	-	15.3
Model’s R-squared	90.7	86.7	91.5	89.7

## Data Availability

The original contributions presented in the study are included in the article/Supplementary Material; further inquiries can be directed to the corresponding authors.

## Supplementary Materials

The following supporting information can be downloaded at: https://www.mdpi.com/article/10.3390/biotech13040054/s1, Figure S1: Infrared-assisted extraction system: schematic and experimental setup. Figure S2: MIC of WB and IR kiwi peel extracts: (a) WB extract on *B. cereus*, (b) IR extract on *B. cereus*, and (c) IR extract on *S. aureus*. Figure S3: HPLC chromatograms of the (a) WB and (b) IR kiwi peel extracts.
