# Supplementary material for: Optimized Extraction of Polyphenols from Kiwifruit Peels and Their Biological Activities
Source: BioTech (Basel). 2024 Dec 2;13(4):54. doi: 10.3390/biotech13040054 (PMC11674650; doi:10.3390/biotech13040054)
Supplement: Supplementary file 1 [file biotech-13-00054-s001.zip › biotech-3268382-supplementary.pdf]

BioTech MDPI

Supplementary material

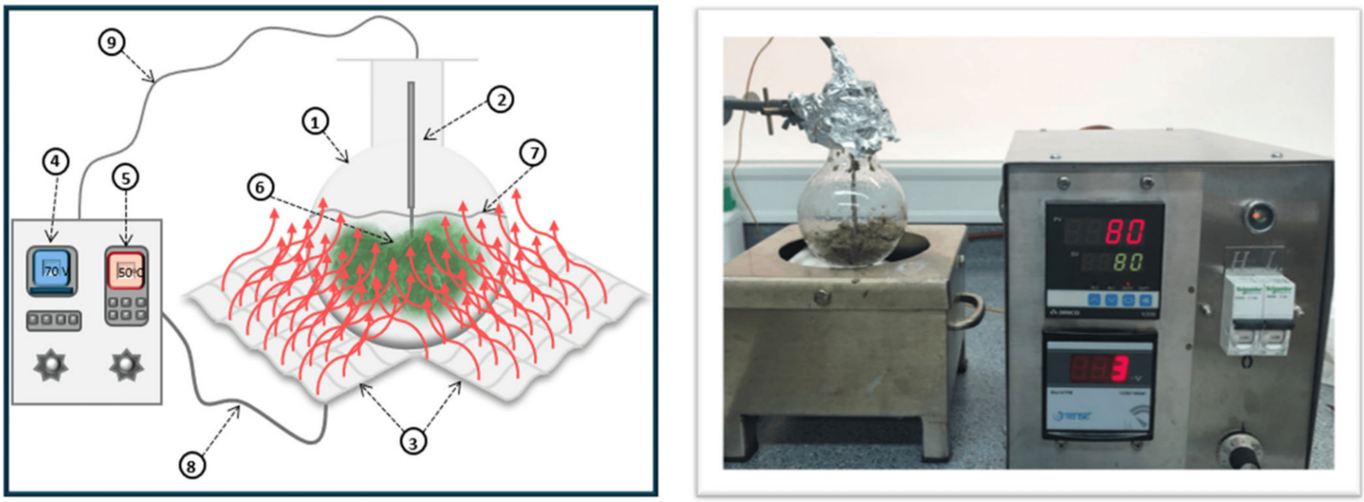

Ired-Irrad pilot : 1) Glass balloon, 2) Thermocouple, 3) Ceramic or quartz infrared emitters, 4) PID voltage modulator, 5) Temperature controller, 6) Product, 7) Solvent mixture, 8) Electric current cable, 9) Thermocouple connection

Figure S1: Infrared-assisted extraction system: schematic and experimental setup.

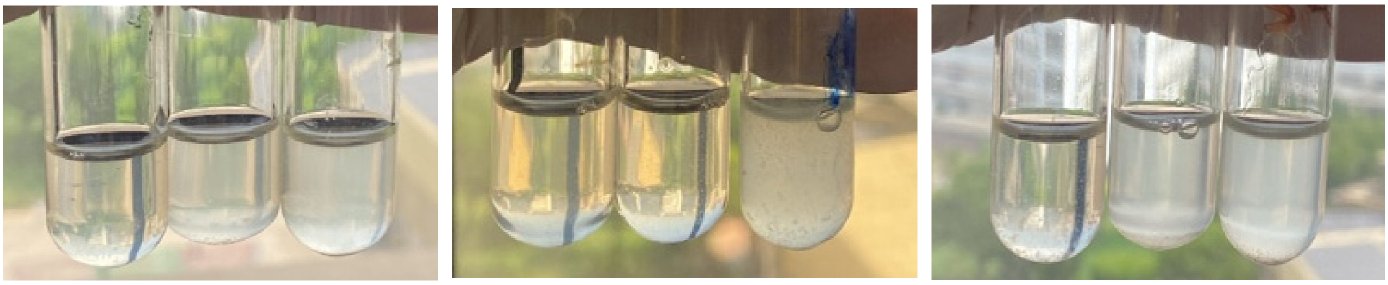

(a) (b) (c)

Figure S2: MIC of WB and IR kiwi peel extracts: (a) WB extract on *B. cereus*, (b) IR extract on *B. cereus*, and (c) IR extract on *S. aureus*. The tubes show the concentration series of each extract, with clear solutions indicating the MIC, or the lowest concentration where bacterial growth is visibly inhibited.

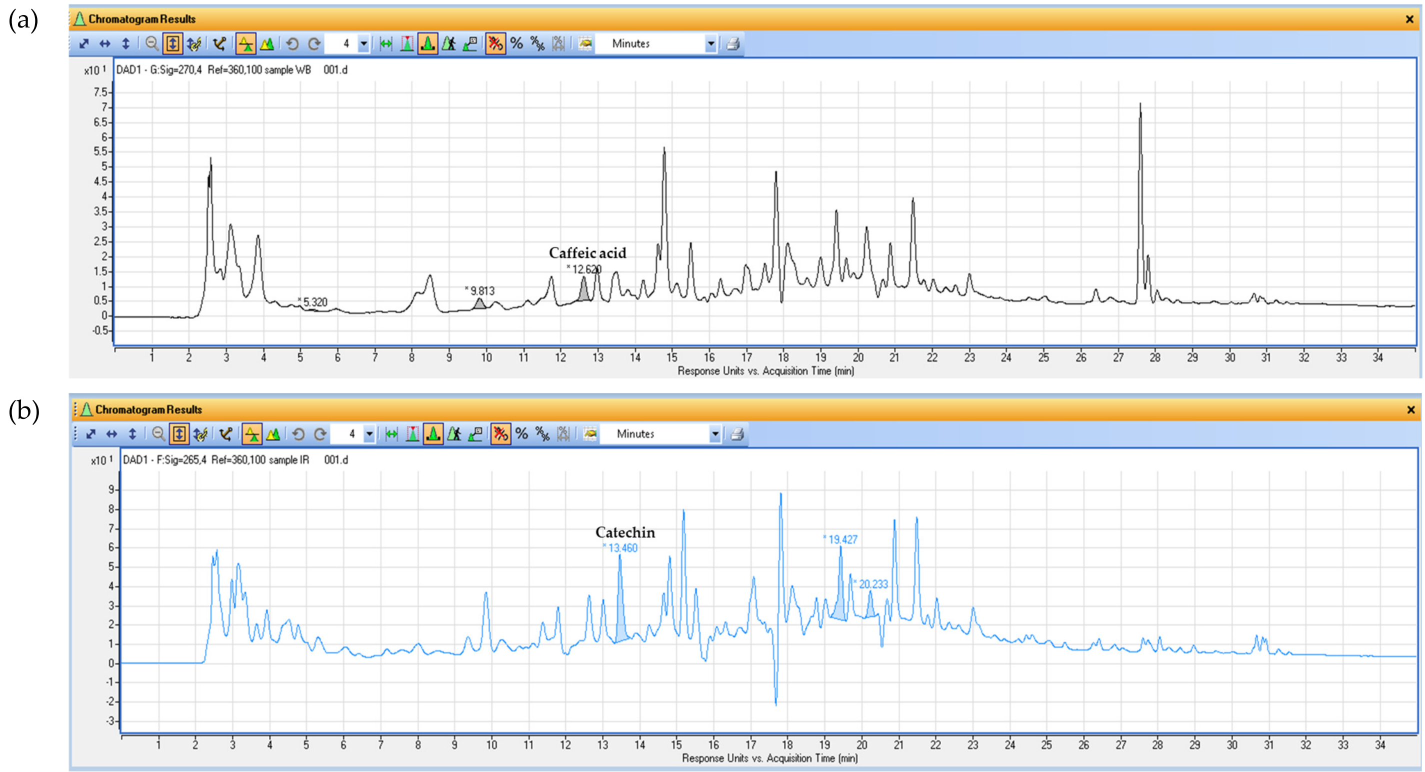

Figure S3: HPLC chromatograms of the (a) WB and (b) IR kiwi peel extracts.
